# Targeting alveolar macrophages shows better treatment response than deletion of interstitial macrophages in EGFR mutant lung adenocarcinoma

**DOI:** 10.1002/iid3.293

**Published:** 2020-03-03

**Authors:** Kristina Alikhanyan, Yuanyuan Chen, Simone Kraut, Rocio Sotillo

**Affiliations:** ^1^ Department of Molecular Thoracic Oncology German Cancer Research Center Heidelberg Germany; ^2^ Translational Lung Research Center Heidelberg (TRLC) German Center for Lung Research (DZL) Heidelberg Germany

**Keywords:** alveolar macrophages, clodronate liposomes, EGFR mutation, interstitial macrophages, lung cancer

## Abstract

**Introduction:**

Alveolar macrophages (AMs) are critical in the development of lung adenocarcinoma driven by epidermal growth factor receptor (EGFR) mutations. Whether interstitial macrophages (IMs) are also involved in lung tumorigenesis is still unclear. Thus, the aim of this study is to evaluate the role of both AM and IM in the development of EGFR mutant driven lung adenocarcinoma.

**Methods:**

We used the EGFR mutant doxycycline‐inducible mouse model of lung adenocarcinoma to deplete interstitial or AMs by clodronate‐encapsulated liposomes administered intravenously (IV) and intratracheally (IT), respectively. Tumor burden, AMs, and the tumor microenvironment were examined by immunohistochemistry, bronchoalveolar lavage fluid or flow cytometry.

**Results:**

Clodronate treatment resulted in a significant reduction of tumor burden compared with vehicle liposomes alone. Elimination of AMs resulted in a significant reduction of proliferation compared with IV treatment. However, both treatments resulted in a significantly higher number of Ki67 positive cells compared with control mice, suggesting that tumor cells still proliferate despite the treatment. The number of natural killer cells decreased during tumor development, and it remained low even after the elimination of AMs. We also observed that IT instillation of clodronate significantly increased the number of CD8+ T cells, which was higher compared with vehicle‐treated mice and mice where only IMs were depleted. The similar trend was observed in immunohistological analyses of CD8+ T cells.

**Conclusions:**

These results suggest that the reduction of AMs has a stronger impact on restricting tumor progression compared with targeting IMs. The depletion of AMs leads to an elevated infiltration of CD8+ T cells into the lung that might be responsible for tumor growth impairment. Altogether, elimination of AMs is a better strategy to reduce EGFR mutant tumor growth and is less toxic, suggesting the selectively targeting of AMs to complement established therapies.

## Graphical abstract

We use a well‐characterized mouse model of epidermal growth factor receptor (EGFR) mutant lung adenocarcinoma and show that depletion of alveolar macrophages (AMs) has a strong impact on restricting lung tumor progression. We further show that the ablation of interstitial macrophages has a milder effect on tumor growth. In addition, we show that depletion of AMs leads to an increased infiltration of CD8+ T lymphocytes that might be responsible for the reduction of tumor growth.

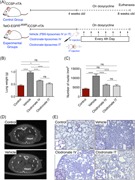

**Abbreviations**
AMs: alveolar macrophages
IMs: interstitial macrophages
IT: intratracheal
IV: intravenous
NSCLC: non–small cell lung cancer

## 1 INTRODUCTION

Fifteen percent of lung adenocarcinomas are driven by epidermal growth factor receptor (EGFR) mutations.^1^ Targeted therapy revolutionized the treatment of EGFR mutant non–small cell lung cancer (NSCLC) with improved response rates over standard chemotherapy, and has become the standard first‐line treatment for patients harboring EGFR mutations.^2^ However, resistance often occurs^3^ due to which the 5‐year survival rate of patients carrying EGFR mutations almost did not change.^4^ Therefore, there is an urgent need to develop new strategies to improve long‐term outcomes for this disease.

During the past decades, many researchers have shifted their focus from the malignant cancer cell itself to the tumor microenvironment and their complex interactions. Macrophages are one of the dominating immune cells in the tumor microenvironment and in response to different stimuli, they can alter their phenotypes having extremely different effects on tumorigenesis.^5^ There are two main types of macrophages: the classically activated M1 which are involved in the inflammatory response and antitumour immunity and M2 that perform anti‐inflammatory and protumorigenic activities.^6^ Tumor‐associated macrophages closely resemble the M2‐polarized macrophages and their density negatively correlates with NSCLC patient survival.^7^ Besides their phenotype, lung macrophages are classified based on their anatomical location into three types: alveolar macrophages (AMs), interstitial macrophages (IMs), and intravascular/marginated vascular macrophages.^8^

Oncogenic EGFR signaling results in the expansion of an overwhelming number of AMs with an immunosuppressive phenotype, and their selective depletion results in a dramatic decrease of tumor burden.^9^ Nevertheless, the role of IMs in the progression of EGFR mutant lung adenocarcinoma remains undefined and is not known if the reduction of IMs could have an increased effect on tumor burden reduction compared to AMs.

## 2 METHODOLOGY

### 2.1 Animals

All animal studies were performed at the DKFZ animal facilities, with ethical approval from the corresponding Animal Welfare and Ethical Review Bodies and national and European legislation. Bitransgenic tetracycline‐inducible human EGFR^L858R^ (TetO‐EGFR^L858R^) animals together with a reverse tetracycline transactivator (rtTA) driven by the Clara cell secretory protein (CCSP) promoter were used.^10^ Control group carried only CCSP‐rtTA. When mice were 4 weeks old, they were fed with doxycycline‐impregnated food pellets (625 ppm; Harlan Laboratories) to express the EGFR mutant transgene. TetO‐EGFR^L858R^ and CCSP‐rtTA transgenic mice were genotyped as described by Politi et al.^10^ Only mice with proven expression of EGFR^L858R^ in lung tissue by quantitative polymerase chain reaction were included in the study (data not shown).

### 2.2 Treatments

**Figure 1:**
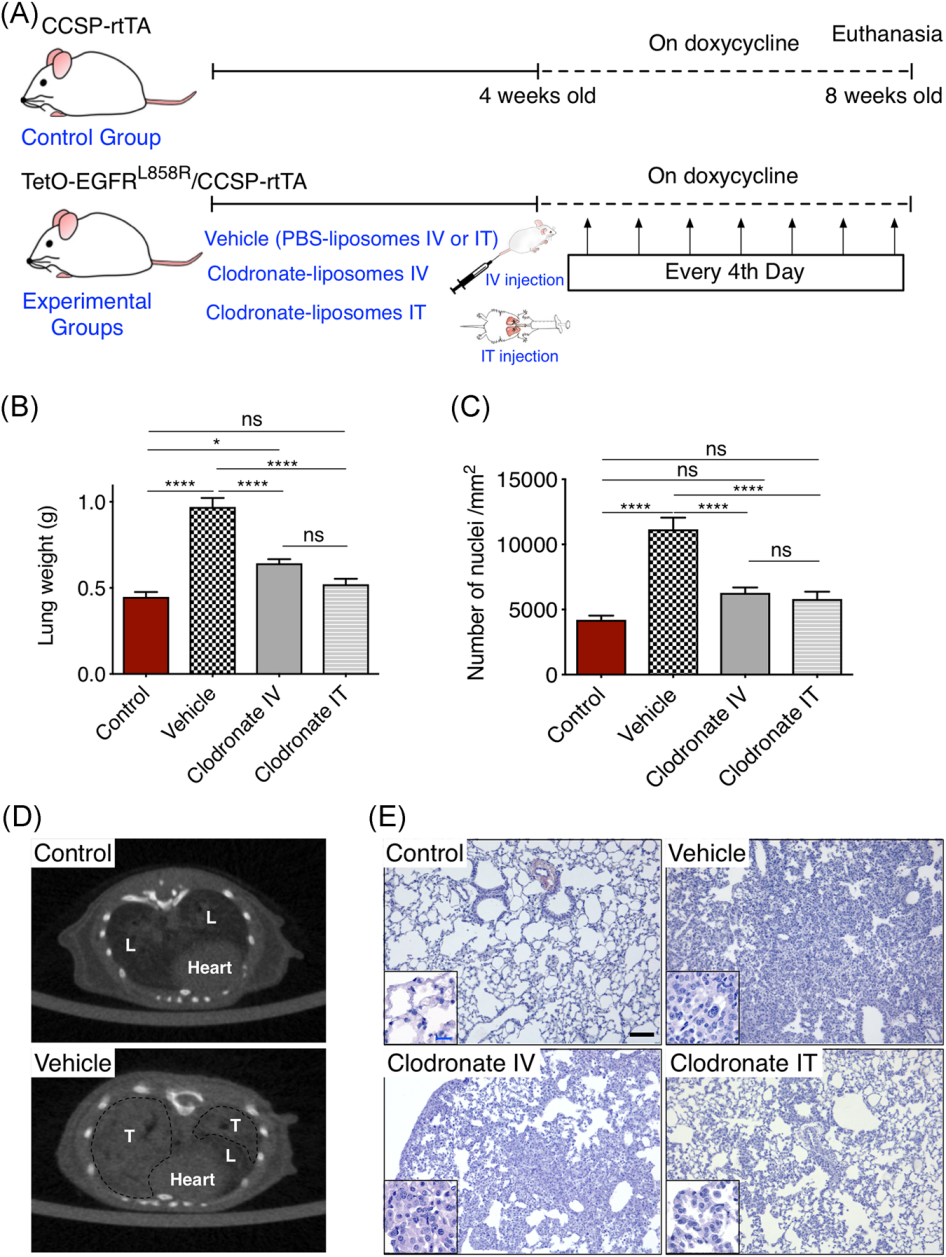
A, Schematic representation of the experiment. Control group carries only CCSP‐rtTA. Experimental groups of bitransgenic TetO‐EGFR^L858R^/CCSP‐rtTA animals were randomized to receive either PBS‐liposomes IV or IT (vehicle group); clodronate‐liposomes IV (clodronate IV group) or IT (clodronate IT group). When mice were 4 weeks old, all groups were fed with doxycycline‐impregnated food for 30 days and were euthanized at 8‐week‐old age. During the doxycycline‐receiving period, every 4th day PBS‐liposomes IV and IT, clodronate‐liposomes intravenously and intratracheally were given to vehicle group, clodronate IV group and clodronate IT group, respectively. B, Tumor burden of control and experimental groups of mice with EGFR^L858R^‐driven lung adenocarcinoma, measured by lung weight and (c) number of nuclei per mm^2^. Data are presented as mean ± SEM (control, n = 5; vehicle, n = 8; clodronate IV, n = 8; clodronate IT, n = 7). *P < .05, **P < .01, ****P < .001 (one‐way ANOVA, Tukey's multiple comparisons). D, Representative micro‐CT images of control mice (upper panel) showing the normal lung tissue (L) and vehicle‐treated mice (lower panel) with tumors (T). E, Representative histological sections of the lungs stained with hematoxylin and eosin. Scale bar = 100 μm, insert 20 μm. ANOVA, analysis of variance; CCSP‐rtTA, Clara cell secretory protein‐reverse tetracycline transactivator; CT, computed tomography; EGFR, epidermal growth factor receptor; IT, intratracheal; IV, intravenous; PBS, phosphate buffer saline

Clodronate (dichloromethylene diphosphonate) encapsulated liposomes or phosphate buffer saline (PBS) control liposomes were purchased from http://www.clodronateliposomes.org. To deplete IMs, when mice started receiving doxycycline food (4 weeks old), 10 µL/g clodronate‐liposome or vehicle (PBS control liposomes) were administered via intravenous route every 4th day for 30 days. To eliminate AMs, under brief anesthesia, a bolus (50 µL) of a suspension of liposome‐encapsulated clodronate or vehicle was injected intratracheally (IT) every 4th day for 30 days (Figure 1A).

### 2.3 Histology

Mice were euthanized by cervical dislocation per institutional guidelines. Lungs were cleared of circulating blood cells by perfusion of the right ventricle with PBS, and the whole lungs were excised and embedded in paraffin. Lung tissues were cut into 5 μm sections and stained with hematoxylin and eosin to assess the lung pathological changes using light microscopy. The number of nuclei was counted using the StrataQuest Analysis Software.

Antibodies against CD8 T cells (1:100; 14‐0808‐80; Thermo Fisher Scientific) and Ki67 (ready‐to‐use; 275R‐18; Medac) were used for immunohistochemical analysis. Ten regions of interest of 1 mm^2^ each per section were analyzed (Figure S2). The sections were counterstained with hematoxylin. Images were acquired with a TG3‐951I TissueFAXS‐i‐plus (fluorescence and bright‐field) system for the scanning and analysis of slides and analyzed with StrataQuest Analysis Software. Bars indicate magnifications.

### 2.4 Collection of bronchoalveolar lavage fluid

The trachea was cannulated and ice‐cold 0.6 mM ethylenediaminetetraacetic acid in PBS (1 mL) was instilled slowly into the lungs. The suspension was then removed and the washout was repeated eight times. The bronchoalveolar lavage fluid (BALF) was centrifuged for 10 minutes at 2000 rpm and the cell pellets were pooled and resuspended in 1 mL of PBS. The total number of living cells in BALF was counted with a Cellometer using trypan blue staining. Hundred cells were analyzed by fluorescence‐activated cell sorting (FACS) for AMs and the percentage was extrapolated to a total number of AMs in the BALF.

### 2.5 Flow cytometry

Whole lungs were dissociated into single‐cell suspensions using the gentleMACS Dissociator. Red blood cell lysing buffer (Sigma‐Aldrich) was used for red cell lysis.

Lung cells were blocked with anti‐mouse CD16/32 (101319; Biolegend) and then stained with antibodies PerCP anti‐mouse/human CD11b (101229; Biolegend), Brilliant Violet 421 anti‐mouse F4/80 (123137; Biolegend), APC/Cy7 anti‐mouse CD45 (103116; Biolegend), PerCP/Cy5.5 anti‐mouse CD11c (117328; Biolegend), PE Siglec‐F (552126; BD Biosciences), PerCP/Cy5.5 anti‐mouse CD4 (100540; Biolegend), PE/Cy7 anti‐mouse CD3ε (100320; Biolegend), Brilliant Violet 421 anti‐mouse CD335 (NKp46) (137612; Biolegend), APC anti‐mouse CD8a (100712; Biolegend), Brilliant Violet 421 anti‐mouse Ly‐6G/Ly‐6C (Gr1) (108433; Biolegend) and Zombie Aqua Fixable Viability Kit (423102; Biolegend). Flow cytometric data acquisition was performed on BD FACS Canto II machine and data analysis was performed using FlowJo software.

Gating for CD45+AquaZombie−Siglec‐F+CD11c+Gr1− cells was used for AMs; CD45+AquaZombie−F4/80+CD11b+Gr1− for IMs CD45+AquaZombie−NKp46+ for natural killer (NK) cells, CD45+AquaZombie−CD3+CD8+ for CD8 T cells.

### 2.6 Micron‐scale computed tomography

Micro‐computed tomography (CT) examinations were performed on a special small animal scanner (Inveon PET/SPECT/CT; Siemens) in the Core Facility Small Animal Imaging of DKFZ. The mice were anesthetized with sevoflurane (2.5% vol/vol) for immobilization. The total anesthetic duration per mouse was less than 60 minutes.

### 2.7 Statistical analysis

Statistical significance was calculated by one‐way analysis of variance (ANOVA). Data were analyzed using GraphPad Prism 8 (GraphPad software). We considered all *P* values .05 as significant. The data shown in each figure represent the mean of three or more independent experiments.

## 3 RESULTS

**Figure 2:**
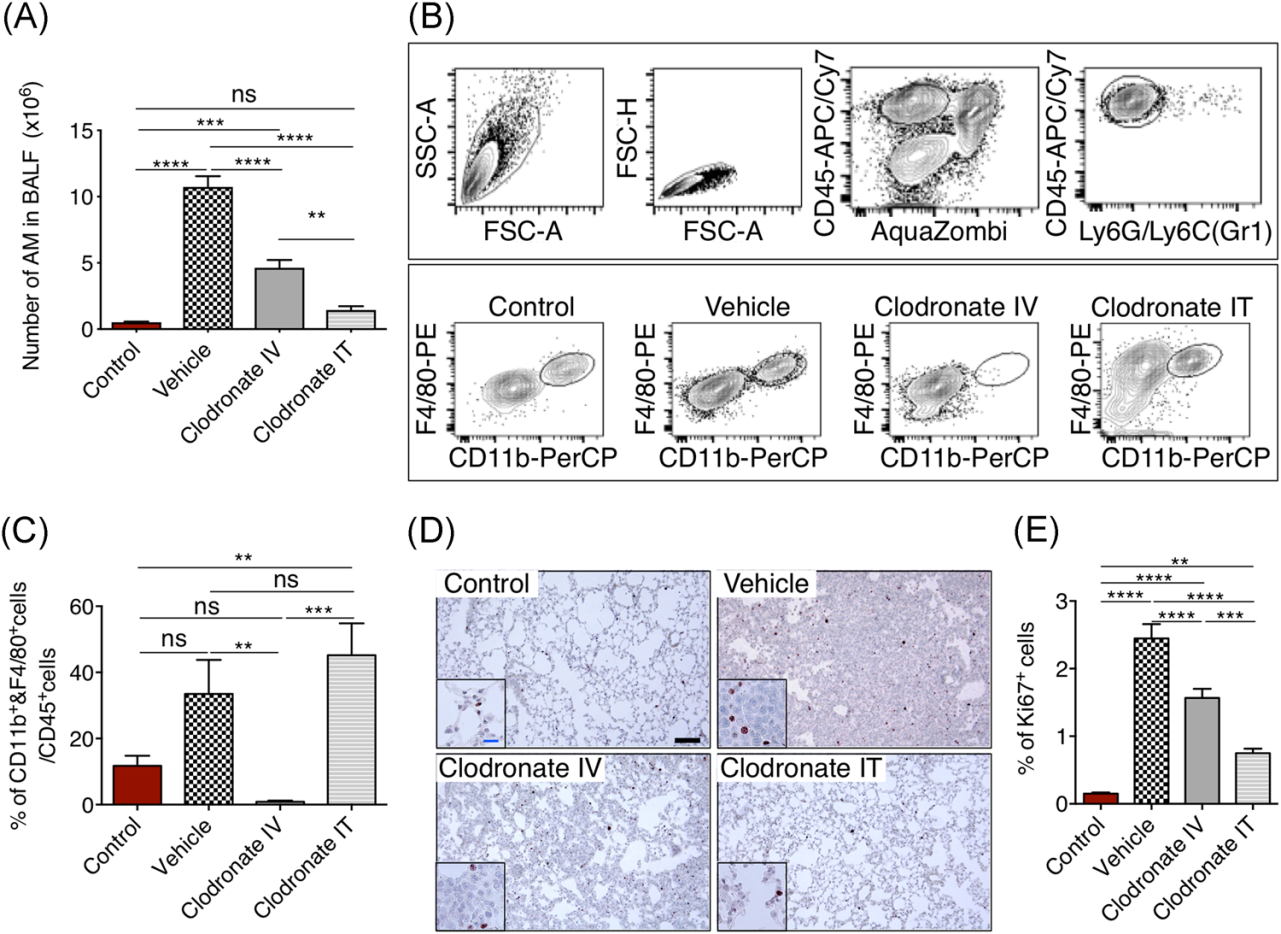
A, Absolute number of AMs in bronchoalveolar lavage fluid (BALF). B, Representative flow cytometry plots of the gating strategy, (upper panel), live CD45+ and Gr1− were then analyzed for CD11b and F4/80 expression (lower panel). Gates were set based on background staining in fluorescence minus one controls. C, Data from three independent experiments are summarized as mean ± SEM and plotted in the bar graph (control, n = 5; vehicle, n = 8; clodronate IV n = 8; clodronate IT, n = 7). *P < .05, **P < .01, ****P < .001 (one‐way ANOVA, Tukey's multiple comparisons). D, Representative histological sections of the lungs showing Ki67 positive cellular staining. Scale bar = 100 μm, insert 20 μm. E, Percentage of Ki67+ cells. Ten regions of interest of 1 mm^2^ per mouse were analyzed with a minimum of 100.000 cells counted per condition. Values represent mean ± SEM; n = 4 mice per group. AM, alveolar macrophage; ANOVA, analysis of variance; IT, intratracheal; IV, intravenously

Taking advantage of an animal model in which mutant EGFR is expressed in the lung after doxycycline exposure,^10^ we studied the role of macrophages during tumorigenesis by using clodronate‐encapsulated liposomes, an efficient reagent for the selective reduction of macrophages. To exclusively deplete AMs, we administered clodronate IT to avoid systemic circulation. For the systemic depletion of myeloid cells in the lung, bone marrow, liver, spleen, and other tissues clodronate liposomes were given intravenously (IV)^11^ (Figure 1A). To test whether AMs and IMs are equally involved in tumor growth and survival, we assessed tumor burden in mice both by lung weight (Figure 1B) and the number of nuclei/mm^2^ (Figure 1C). EGFR mutant mice fed with doxycycline food for 30 days and receiving vehicle liposomes presented a dramatic increase in tumor burden compared with control animals which did not express mutant EGFR, shown by increased lung weight (Figure 1B), increased number of nuclei/mm^2^ (Figure 1C) or micro‐CT scan of the entire lung (Figure 1D). Interestingly, 4 weeks of clodronate treatment IT as well as IV resulted in a significant reduction of tumor burden compared with vehicle liposomes alone, though the lowest tumor burden was observed in IT‐treated mice (Figures 1B,C,E and S1). To further ascertain that clodronate treatment reduced the number of macrophages, we counted the absolute number of AMs in the BALF and performed FACS analyses of IMs. As already described by Wang et al^9^ the number of AMs increased significantly when mice were fed doxycycline for 4 weeks from an average of 0.5136 million AMs in the lungs of control mice (no EGFR expression) to 10.73 million (EGFR mutant expressing animals) (Figure 2A). As expected, IT injection of clodronate significantly reduced AMs (1.439 million) while IV administration resulted also in a significant reduction of macrophages (4.635 million), although less pronounced. In addition, the number of IMs (Figure 2B,C) was much lower in the IV‐treated group compared with the other groups. Importantly, the percentage of IMs was high in vehicle‐treated animals and it stayed at the same level in IT‐treated ones, despite the reduction in tumor burden. Next, to better understand whether the reduction of macrophages by clodronate treatment resulted only in the killing of tumor cells or whether it had an effect on proliferation, we measured the percentage of Ki67 stained cells among the different groups (Figures 2D,E and S2). As expected, the percentage of Ki67 positive cells in the samples with continuous EGFR signaling (vehicle liposomes) was significantly higher (2.45% ± 0.214%) than in the control group (0.15% ± 0.017%) (Figure 2E). Elimination of AMs by IT treatment of clodronate resulted in a significant reduction in Ki67 positive cells (0.75% ± 0.068%) compared with IV treatment (1.57% ± 0.136%), which can explain the lower tumor burden in IT‐treated group compared with IV‐treated one. Notably, both treatments resulted in a significantly higher number of Ki67 positive cells compared with control mice, suggesting that tumor cells still proliferate despite the treatment (Figure 2D,E), although significantly less than in a vehicle‐treated group.

**Figure 3:**
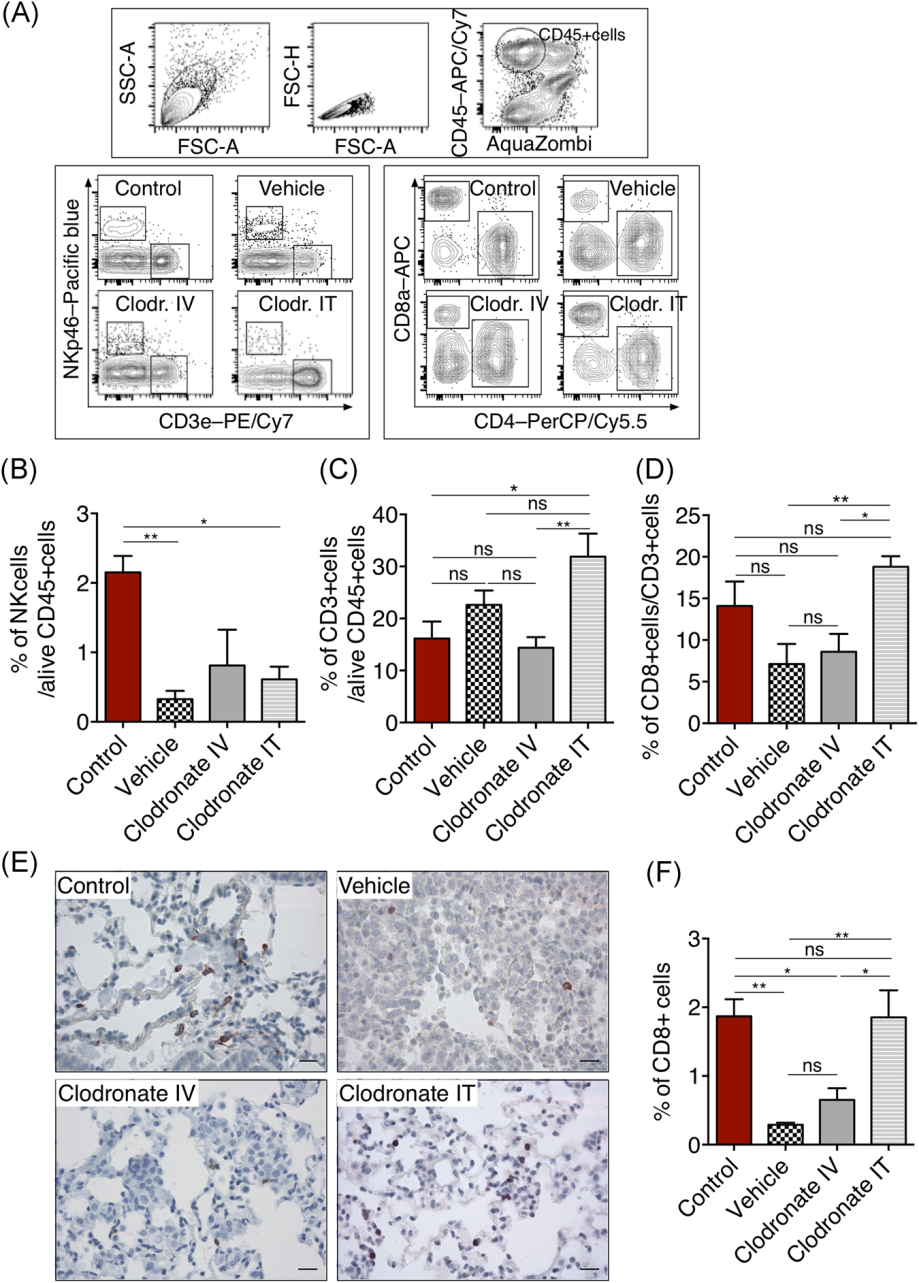
A, Representative flow cytometry plots of the gating strategy, (upper panel), live CD45+ cells were then analyzed for NKp46 and CD3 (lower left panel) or for CD8 and CD4 cells (lower right panel). B, Percentage of NKp46+ cells out of alive CD45+ cells. C, Percentage of CD3+ T cells in alive CD45+ cells and (D) percentage of CD8+ T cells out of CD3+ T cells. E, Representative images of lung tissues stained for anti‐CD8. Scale bar = 20 μm. F, Percentage of CD8+ T cells. Values represent mean ± SEM; n = 4 mice per group. All data in B‐D are presented as mean ± SEM (control, n = 5; vehicle, n = 8; clodronate IV n = 8; clodronate IT, n = 7); *P < .05, **P < .01, ***P < .001 (one‐way ANOVA, Tukey's multiple comparisons). ANOVA, analysis of variance; IT, intratracheally; IV, intravenously; NK, natural killer

To characterize the immune landscape of lung tissues after the elimination of AMs or IMs, we performed flow cytometry analysis of NK cells, CD3+ T cells, and CD8+ T cells. As shown in Figure 3A,B, the number of NK cells decreased during tumor development, and it remained low even after the elimination of AMs. However, we observed that IT instillation of clodronate significantly increased the number of T cells (Figures 3A and 3C), mainly CD8+ T cells, which was significantly higher compared with vehicle‐treated mice and mice where only IMs were depleted (Figure 3D). To validate these data, we further analyzed CD8+ T cells by immunohistochemistry (Figure 3E,F) and observed a similar trend, suggesting that elimination of AMs indeed increased cytotoxic CD8+ T‐cell infiltration in the lung, which might explain the lower tumor burden of this group. Altogether, elimination of AMs suggests a better strategy to reduced EGFR mutant tumor growth and in addition less toxic since IV injection of clodronate was accompanied by pathological changes in spleen and liver (Figure S3A‐D), hinting its high toxicity.

Our data show that AMs play a more potent role in driving EGFR mutant lung adenocarcinoma development compared with IMs. Moreover, depletion of AMs in the lung results in an elevated number of CD8+ T‐cell infiltration, tumor cell killing, and better prognosis after treatment. Therefore, selectively targeting alveolar rather than IMs will help to progress the development of specific AM‐targeted therapies which can add complementary benefits to established therapies.

## CONFLICT OF INTERESTS

The authors declare that there are no conflict of interests.

## Supporting information

Supporting information

Supporting information

Supporting information

Supporting information

## Data Availability

All data is shown within the manuscript, figures, and Supporting Information.
