# Supplementary figures and images for: Targeting alveolar macrophages shows better treatment response than deletion of interstitial macrophages in EGFR mutant lung adenocarcinoma
Source: Immun Inflamm Dis. 2020 Mar 3;8(2):181–7. doi: 10.1002/iid3.293 (PMC7212197; doi:10.1002/iid3.293)

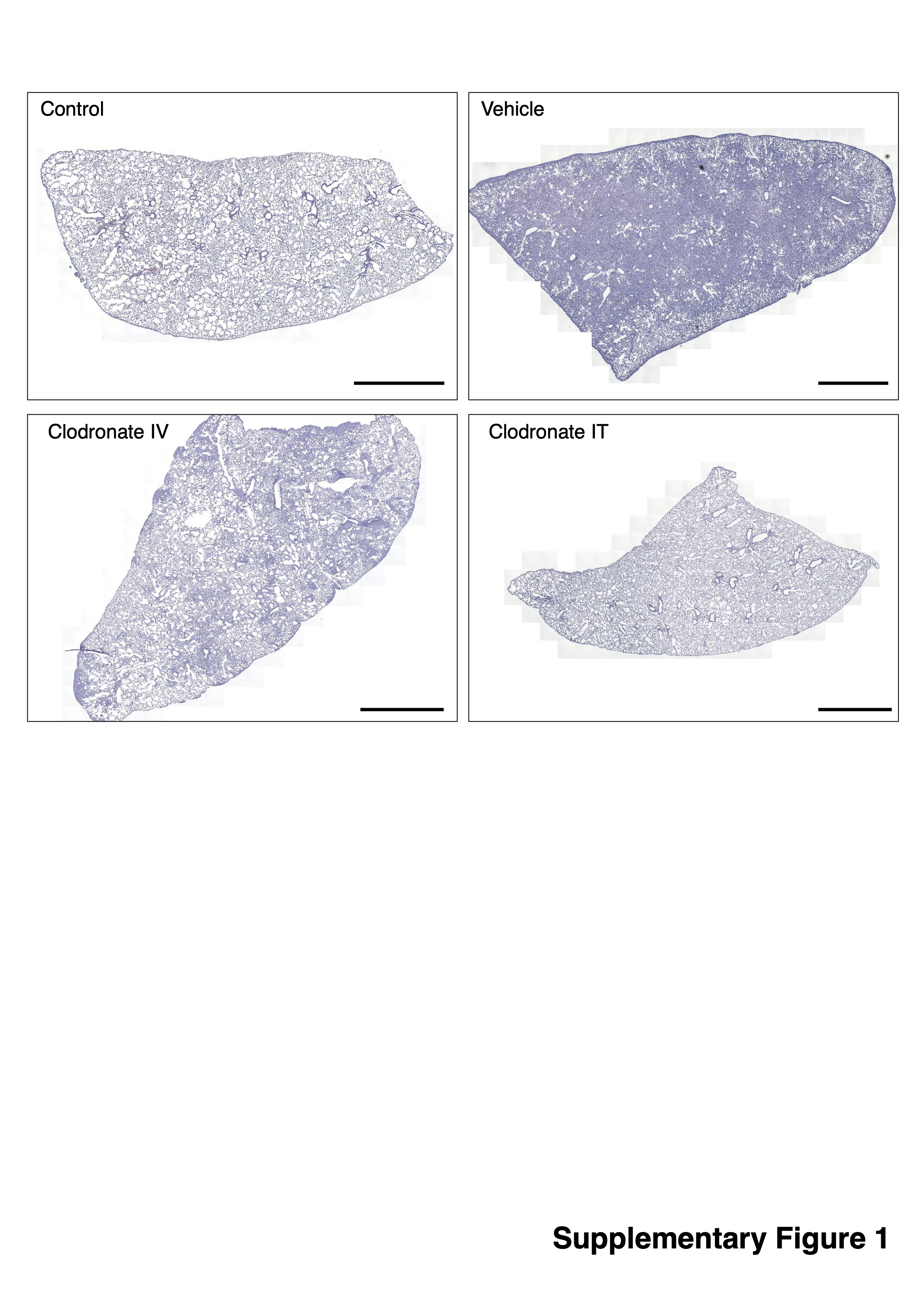

Supplement: Supplementary file 1 — Supporting information [file IID3-8-181-s001.tiff]

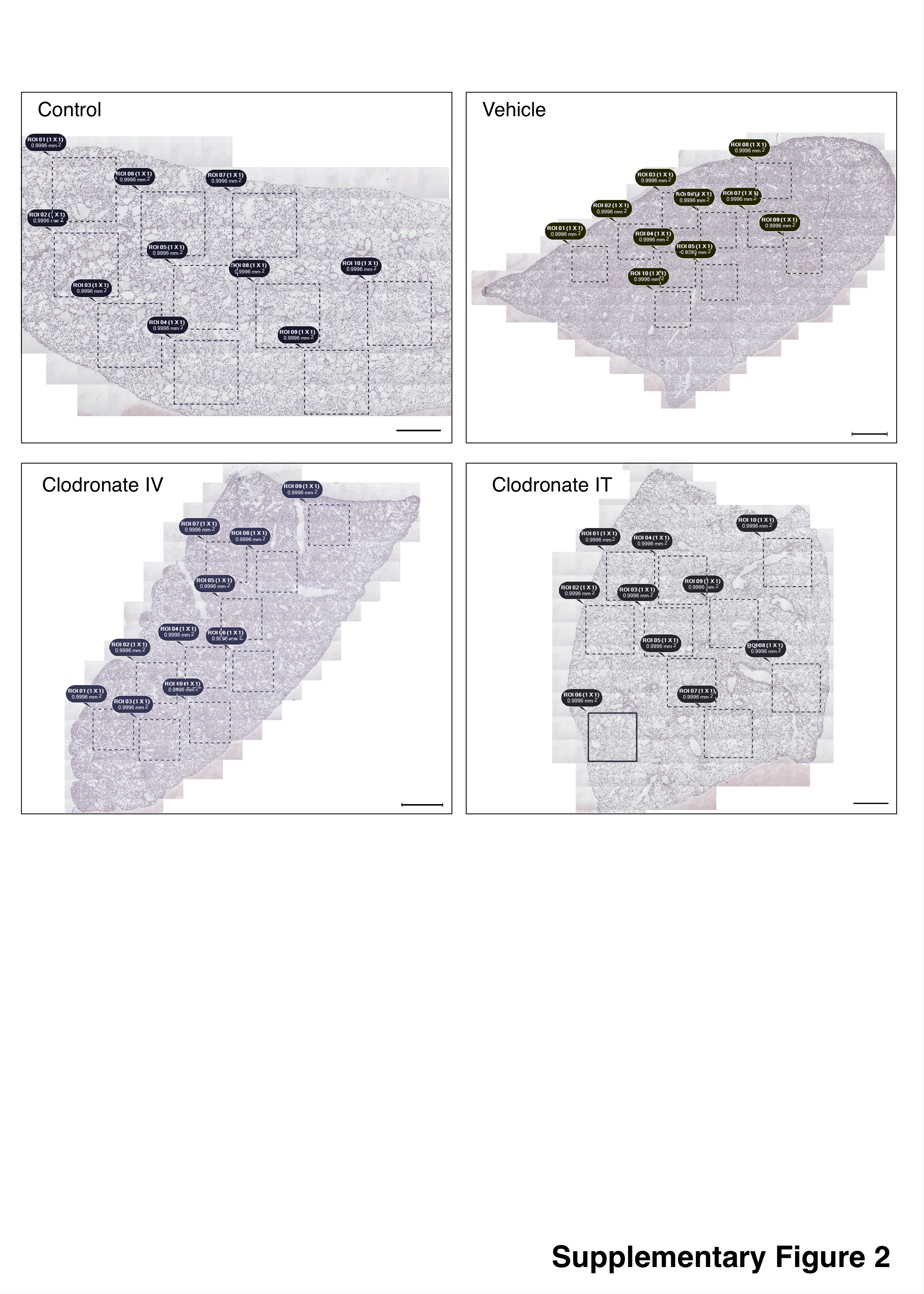

Supplement: Supplementary file 2 — Supporting information [file IID3-8-181-s002.tiff]

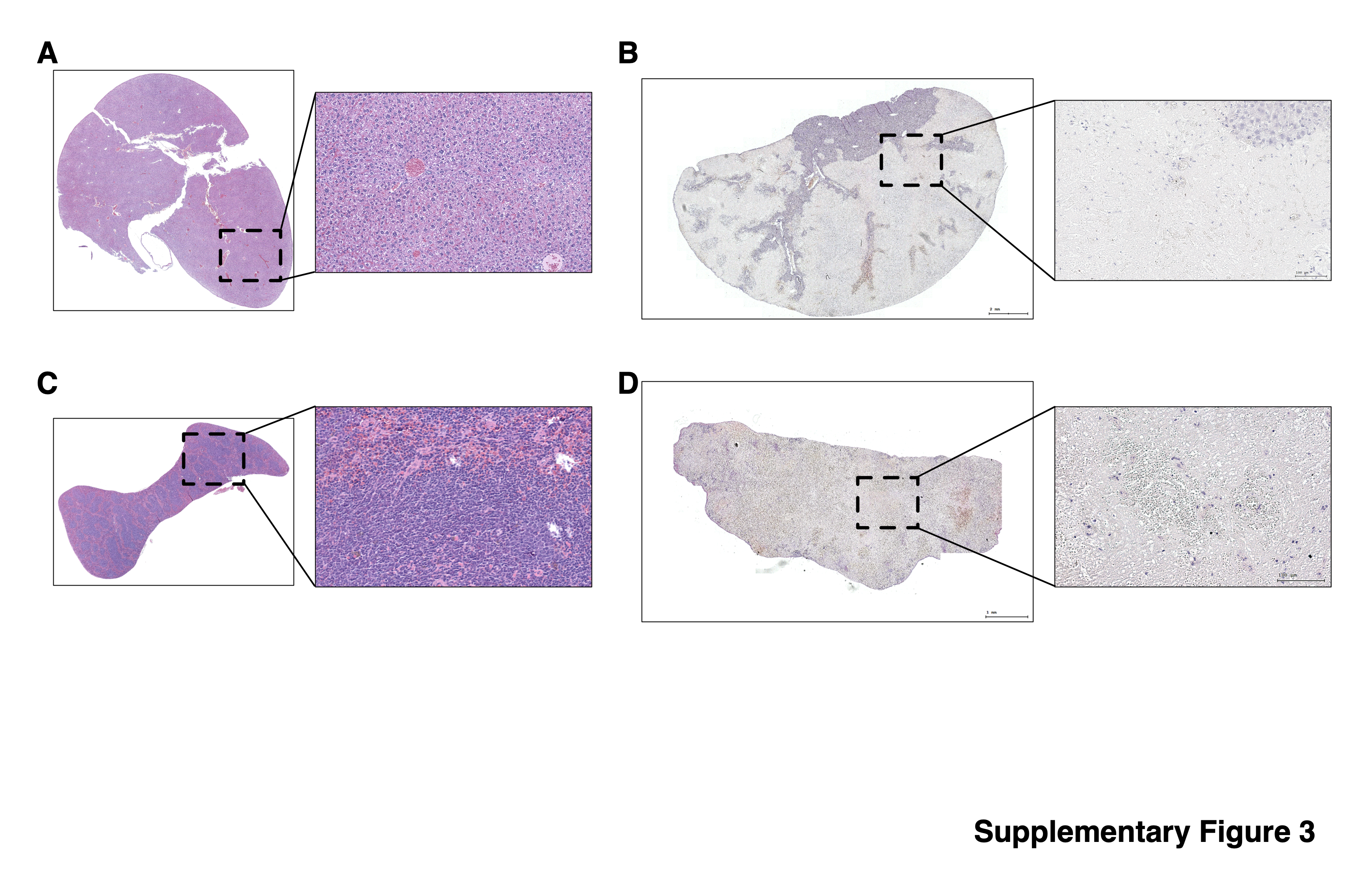

Supplement: Supplementary file 3 — Supporting information [file IID3-8-181-s003.tiff]
