# Supplementary material for: Targeting alveolar macrophages shows better treatment response than deletion of interstitial macrophages in EGFR mutant lung adenocarcinoma
Source: Immun Inflamm Dis. 2020 Mar 3;8(2):181–7. doi: 10.1002/iid3.293 (PMC7212197; doi:10.1002/iid3.293)
Supplement: Supplementary file 4 — Supporting information [file IID3-8-181-s004.docx]

**Supplementary figure legends:**

**Supplementary Figure 1.** Representative whole-slide images of lung sections, stained with hematoxylin and eosin. Scale bar, 1mm.

**Supplementary Figure 2.** Representative whole-slide images of lung sections, stained with Ki67. 10 random ROIs were selected in each section for further analyses.

**Supplementary Figure 3.** Representative whole-slide images and insert of normal liver (**A**) and normal spleen (**C**). (**B**) Representative whole-slide images and insert of the liver of Clodronate IV treated mice and spleen (**D**).
